# International disparities in use of antenatal magnesium sulfate and antenatal steroids for the preterm baby

**DOI:** 10.1002/ijgo.70832

**Published:** 2026-02-25

**Authors:** Hannah B. Edwards, Erika M. Edwards, David Odd, Jelena Savović, Sarah Dawson, Mark Adams, Angelika Berger, Paul Corcoran, Jose Maria de Andrade Lopes, Luigi Gagliardi, R. Kishore Kumar, Rajesh Sharma, Lloyd Tooke, Frank de Vocht, Karen Luyt

**Affiliations:** ^1^ Population Health Sciences, Bristol Medical School University of Bristol Bristol UK; ^2^ The National Institute for Health and Care Research Applied Research Collaboration West (NIHR ARC West) at University Hospitals Bristol and Weston NHS Foundation Trust Bristol UK; ^3^ College of Engineering and Mathematical Sciences University of Vermont Burlington Vermont USA; ^4^ Vermont Oxford Network (VON) Burlington Vermont USA; ^5^ Population Medicine, Cardiff University School of Medicine Cardiff University Cardiff UK; ^6^ Neonatology, Cardiff and Vale University Health Board Cardiff UK; ^7^ Department of Neonatology University Hospital Zurich, and University of Zurich Zurich Switzerland; ^8^ Department of Pediatrics and Adolescent Medicine Medical University Vienna Vienna Austria; ^9^ Department of Obstetrics and Gynecology University College Cork, and Cork University Maternity Hospital Cork Ireland; ^10^ Brazilian Neonatal Research Network Institute São Paulo Brazil; ^11^ Ospedale Versilia, Department of Woman and Child Health AUSL Toscana Nord Ovest Pisa Italy; ^12^ Neonatology, Cloudnine Group of Hospitals and Cloudnine Foundation Chennai India; ^13^ Department of Neonatology Corniche Hospital Abu Dhabi UAE; ^14^ Department of Neonatology University of Cape Town Cape Town South Africa; ^15^ Translational Health Sciences, Bristol Medical School University of Bristol Bristol UK; ^16^ Neonatology, St Michael's Hospital Bristol UK

**Keywords:** antenatal magnesium sulfate, antenatal steroids, disparities, evidence‐based interventions, implementation, preterm birth, review

## Abstract

Antenatal magnesium sulfate (MgSO_4_) and antenatal steroids (ANS) are evidence‐based interventions that reduce risk of cerebral palsy and respiratory complications in preterm babies. They are recommended in clinical guidelines internationally. However, we have limited information on how well they are being implemented. The present study is a secondary data analysis and review using routine neonatal data on babies born 24–32 weeks' gestation, from hospitals in the international Vermont Oxford Network (VON) dataset. It is supplemented with UK National Neonatal Research Database data, and a literature review. We describe international use of antenatal MgSO_4_ and ANS, with focus on differences between high (HIC) versus middle‐income (MIC) countries. VON data from 2024 on 45 619 infants across 1111 centers from the UK, Ireland, Austria, Switzerland, Italy, US, UAE, Brazil, South Africa, and India were included. Ireland and the UK had the highest rates of MgSO_4_ administration (>80%); South Africa and the UAE had the lowest (33.6%, 44.5%). There was a significant difference by income status (mean 74.8% in HICs vs. 49.4% in MICs). This disparity does not appear to have reduced over time. ANS were used more, with less variation. The supplementary literature review (10 studies reporting on 288 631 infants) found comparable treatment rates to those reported in VON. Use of antenatal MgSO_4_ and ANS varies considerably across countries. Uptake is significantly lower in these MICs, but variation is high even between these HICs. Further work should prioritize understanding why differences exist, and what can be done to make these key antenatal interventions more globally equitable.

## 1 INTRODUCTION

Babies born preterm have a higher risk of death and disability including intraventricular hemorrhage (IVH), respiratory distress, developmental delay, and cerebral palsy (CP). An updated systematic review in 2024 confirmed strong evidence that administering antenatal magnesium sulfate (MgSO_4_) to the mother in preterm labor is neuroprotective and reduces the risk of CP by around 30%.^1^ A systematic review in 2020 found strong evidence that antenatal steroids (ANS) reduces the risk of perinatal death and respiratory distress, and possibly reduces the risk of IVH and developmental delay.^2^ Both of these antenatal interventions are recommended in guidelines,^3^, ^4^, ^5^, ^6^, ^7^ including the recent International Federation of Gynecology and Obstetrics (FIGO) “PremPrep‐5 initiative,” which details five simple, key interventions to improve neonatal outcomes, and are feasible across high to low income settings.^7^ There is good evidence that both interventions are cost‐effective.^8^, ^9^, ^10^, ^11^, ^12^

However, there are gaps in our knowledge of how well these treatments, particularly MgSO_4_, are being used in practice. Internationally there have been various interventions to improve use of MgSO_4_,^13^, ^14^, ^15^, ^16^, ^17^ and there are isolated reports on local and national use.^13^, ^16^, ^18^, ^19^, ^20^, ^21^, ^22^ Editorials have reported that across hospitals participating in the international VON, described below, overall MgSO_4_ uptake was around 69% in 2022,^23^ up from 46% in 2012.^24^ ANS is a more well‐established antenatal intervention, but there has been debate and concern recently regarding potential longer‐term harms, the highly variable effects in different gestational age (GA) groups, and over‐treatment both by dose and in the population of infants who go on to be born at term.^25^, ^26^ These recent issues indicate that previously high rates of ANS use could be changing. In this context, this study aimed to review how antenatal MgSO_4_ and ANS are currently being used internationally.

## 2 METHODS

### 2.1 Study design

This was a secondary data analysis using routinely, prospectively collected, longitudinal, observational, aggregate‐level data.

### 2.2 Aims

We aimed to describe and review international uptake of antenatal MgSO_4_ and ANS.

### 2.3 Data sources

#### 2.3.1 Vermont Oxford network data

Data was drawn from the VON dataset. VON is an international, voluntary network of hospitals with neonatal intensive care units (NICUs). Its purpose is to improve the quality, safety, and value of neonatal care, via collection of standardized and quality‐controlled data, benchmarking reports, and participation in research and quality improvement (QI) initiatives.^27^ All data were collected and checked by the local NICU clinical and administrative staff using standardized definitions. Participating units absorb the staff time costs of data collection and submission. VON data are considered high‐quality^28^ and high completeness (in general ≤1% missing data on any variable).

VON data used in this study came from participating neonatal centers across Austria, Brazil, Italy, Ireland, India, South Africa, Switzerland, the UAE, the UK, and the US, on live‐born infants between 24 weeks' and 0 days gestation (24^+0^) and 32 weeks' and 6 days gestation (32^+6^) inclusive, with birth weight up to 1500 g, who were admitted to or died at the participating centers within 28 days of birth.^29^ VON data were aggregated and shared by the VON team. This included summary maternal and infant characteristics, with proportions of infants receiving MgSO_4_ and/or ANS, aggregated by country. Data covered the period January 1, 2012 to December 31, 2024.

#### 2.3.2 National Neonatal Research Database data

A secondary data source was the UK National Neonatal Research Database (NNRD), including routinely collected healthcare data on all live‐born infants admitted to a UK National Health Service (NHS) neonatal unit.^30^ NNRD data used in this study was restricted to infants of the same GA (24^+0^–32^+6^ weeks), born in England in 2024.

#### 2.3.3 Other data sources on MgSO_4_ use

As not all countries are represented in VON data, we additionally carried out a supplementary review of the literature for other sources of relevant published data on MgSO_4_ use. Eligibility criteria included: audit reports, QI reports, cohort studies, cross‐sectional studies, and surveys. These needed to be national or at least regional‐level, and report figures based on individual‐level data on MgSO_4_ exposure for infants 24^+0^–32^+6^ weeks' gestation, within the last 10 years (see Appendix S1 for full details).

Relevant reports were identified in two ways. First, we searched for published articles in two bibliographic databases, MEDLINE (Ovid) and CINAHL (EBSCO) using relevant subject headings, keywords, and search syntax appropriate to each resource (see Appendix S2 for an example search strategy). Second, we conducted iterative gray literature searches using Google and forward/backward citation searching, with a narrower focus on national‐level audit reports from high‐income European countries within the last 5 years (2020–2025). The gray literature search was more narrowly defined for several reasons: (a) Pragmatically, gray literature searching is complex, it needs to be focused as it can otherwise become unwieldy. (b) HICs are much more likely to run national maternity and perinatal audits than MICs/LICs, so any existing data is most likely to be found in that group. (c) We already had known ideal data sources from HICs in other continents.

Results were screened by SD and HE, and data extracted from included results by HE. Data extracted included country, year of data reported on, data or study type, number and type of centers, number and age of infants, reported MgSO_4_ uptake, and reported ANS uptake. In cases where relevant data was not directly reported in the source paper, where possible this was either calculated from the data provided (e.g., GA‐specific rates) or obtained from connected sources (e.g., from study authors or other sources referenced in the included paper).

Data synthesis was mainly narrative and descriptive, and we did not perform a risk of bias assessment as the purpose was not to produce summary estimates across studies, but just to describe practices.

### 2.4 Analyses

This was a descriptive study. The primary outcome of interest was national MgSO_4_ uptake, defined as the proportion of eligible babies receiving antenatal MgSO_4_ (number exposed divided by number unexposed, excluding number with missing MgSO_4_ data). All babies in this dataset are considered eligible for treatment. The secondary outcome of interest was national uptake of ANS, defined the same way. Descriptive analysis included tables describing VON hospital and infant characteristics, with the most recent (2024) data on receipt of MgSO_4_ and ANS, aggregated nationally, and figures showing time‐trends in use of MgSO_4_ and ANS.

Supplementary analyses included uptake by GA group and within‐country variation. To evaluate the representativeness of the VON data, VON data from the participating English centers was compared to equivalent aggregated NNRD data on all hospitals in England. MgSO_4_ and ANS uptake as reported in other literature from the supplementary review was described separately.

### 2.5 Ethics

VON data was used with the permission of participating centers. The University of Bristol and VON confirmed that no further ethical or other permissions were indicated.

## 3 RESULTS

### 3.1 VON hospital characteristics

VON‐participating centers in Austria, Brazil, Italy, Ireland, India, South Africa, Switzerland, the UAE, the UK, and the US were included. The number of centers per country ranged from nine in Switzerland to 791 in the US (1111 total centers). Most of these countries are classified as HICs. Brazil and South Africa are upper‐MICs, India is a lower‐MIC (grouped together as MICs). Centers in Austria, Ireland, Switzerland and the UK were entirely not‐for‐profit. Other countries ranged from 2.6% for‐profit hospital ownership in Italy to 90% in India. In the US (contributing the most centers) for‐profit hospital ownership was 15.5% (Table 1).

**TABLE 1:** Hospital characteristics in VON data for 2024.

Country	Number of centers	Income status	For‐profit hospital ownership	Relevant guidelines followed
UK	28	High	0.0%	WHO (2015), NICE (2022)
Ireland	17	High	0.0%	RCPI (2015, 2024)
Austria	10	High	0.0%	AWMF (2023)
Switzerland	9	High	0.0%	SSGO (2019)
Italy	47	High	2.6%	AOGOI (2020)
US	791	High	15.5%	ACOG (2023)
UAE	12	High	75%	CHCG (2023, 2025)
Brazil	53	Upper‐middle	28.6%	FEBRASGO (2025)
South Africa	112	Upper‐middle	86.0%	GMCSA (2016), EML (2019), SASOG (2016)
India	32	Lower‐middle	90.0%	WHO (2015), FOGSI (2025)

Abbreviations: ACOG, American College of Obstetricians and Gynecologists; AOGOI, Association of Obstetricians and Gynecologists of Italy; AWMF, Association of the Scientific Medical Societies in Germany; CHCG, Corniche Hospitals Clinical Guideline — Antenatal Corticosteroids (2023); — Magnesium Sulfate Before Anticipated Preterm Birth for Fetal Neuroprotection (2025) American College of Obstetricians and Gynecologists — Magnesium Sulfate Before Anticipated Preterm Birth for Neuroprotection; EML, Standard Treatment Guidelines and Essential Medicines List for South Africa; FEBRASGO, Brazilian Society of Obstetric and Gynecology Protocol for Fetal Neuroprotection in Preterm Labor; FOGSI: Federation of Obstetrics & Gynecology Society of India — Preterm Labor Practice Algorithms; GMCSA, Guidelines for Maternity Care in South Africa; NICE, National Institute of Health and Care Excellence — Preterm Labor and Birth; RCPI, Clinical practice guidelines from Institute of Obstetricians and Gynecologists, Royal College of Physicians of Ireland — Antenatal Magnesium Sulfate for Fetal Neuroprotection (2015); — Antenatal Corticosteroids to Reduce Neonatal Morbidity and Mortality (2024); SASOG: South African Society of Obstetricians and Gynecologists; SSGO, Swiss Society of Gynecology and Obstetrics; VON, Vermont Oxford Network; WHO: World Health Organization — WHO recommendations on interventions to improve preterm birth outcomes.

### 3.2 VON infant characteristics

From January 1 to December 31, 2024, there was VON data on a total of 45 619 infants 24^+0^–32^+6^ weeks' GA across the countries listed above. Median GA and birth weight were comparable across countries (28–29 weeks' median GA, and 1049 g (UK) to 1160 g (India) birth weight). The proportion of infants defined as small for gestational age (SGA) varied more between countries, from South Africa at the lowest (11.6%) to Brazil at the highest (21.3%). The proportion of multiple births also varied, from South Africa at the lowest (21.7%) to India at the highest (36.9%). The proportion of inborn infants was lowest in the UK (80%) and highest in the UAE (98.1%). The proportion of cesarean deliveries was lowest in the UK (71.9%) and highest in Austria (91.4%). The proportion with maternal hypertension was lowest in the UK (16.5%) and highest in the US (44.3%) (Table 2).

**TABLE 2:** Infant characteristics in VON data for 2024, infants 24^+0^ to 32^+6^ weeks gestational age.

Country	Number of eligible babies	Gestational age, weeks (median, IQR)	Birth weight, grams (median, IQR)	SGA^a^ (%)	Multiple (%)	Inborn (%)	Cesarean delivery (%)	Maternal hypertension (%)
High‐income								
UK	1242	28 (26, 30)	1049 (800, 1300)	13.3	25.8	80.0	71.9	16.5
Ireland	395	29 (27, 30)	1130 (875, 1350)	14.0	27.6	91.1	74.9	31.6
Austria	440	28 (26, 30)	1072 (807, 1312)	15.2	33.6	96.8	91.4	23.3
Switzerland	474	28 (26, 30)	1055 (820, 1285)	15.8	32.1	96.6	83.3	25.2
Italy	1283	29 (27, 31)	1118 (870, 1330)	19.8	25.7	93.8	79.2	27.9
US	34 662	28 (26, 30)	1100 (845, 1320)	14.7	23.1	86.3	77.8	44.3
UAE	538	28 (27, 30)	1100 (850, 1315)	14.3	27.5	98.1	76.6	20.1
Middle‐income								
Brazil	2864	29 (27, 30)	1055 (815, 1280)	21.3	23.7	96.9	74.9	42.8
South Africa	3259	29 (27, 30)	1100 (885, 1300)	11.6	21.7	89.8	73.2	36.6
India	462	29 (27, 31)	1160 (920, 1340)	18.8	36.9	84.4	84.0	25.6

Abbreviations: IQR, interquartile range; PMA, post menstrual age; SGA, small for gestational age; VON, Vermont Oxford Network.

^a^ SGA is coded as “Yes” in VON if the infant's Z‐score for weight at birth is less than the 10th percentile value based on the PMA and sex of infant. This applies to infants whose gestational age at birth was between 22^+3/7^ weeks and 46^+6/7^ weeks and whose birth weight was greater than 200 g. Tenth percentile values are based on the Fenton growth chart.

### 3.3 MgSO4 and ANS uptake in VON centers

Across all countries, ANS were used more often than MgSO_4_. There was a clear treatment gap in use of both treatments between these HICs and MICs, most strikingly for MgSO_4_. The total proportion of infants receiving MgSO_4_ was highest in Ireland and the UK (83.6%, 82%) and lowest in South Africa and the UAE (33.6%, 44.5%), with a treatment gap of around 25 percentage points between these HICs and MICs (mean 74.8% in HICs compared to 49.4% in MICs).

The total proportion of infants receiving ANS was highest in Ireland (93.1%) and lowest in South Africa (73.1%). For ANS there was a smaller treatment gap of around 10 percentage points (mean 88.1% in HICs compared to 77.8% in MICs).

Across all countries, receiving MgSO_4_ only was very rare (around 2%). Receiving ANS only was more common, and a much more frequent outcome in these MICs (31.2%) compared to these HICs (15.6%).

Across most of these countries, >50% of infants received both MgSO_4_ and ANS. The exceptions were South Africa (31.9%) and the UAE (42.9%). The UK and Ireland had the highest proportions of infants receiving both interventions (81%, 82.3%). Again, the mean uptake for both treatments in these HICs and MICs differed significantly (72.6% vs. 47.3%) (Table 3).

**TABLE 3:** Exposure to antenatal interventions in VON data for 2024, infants 24^+0^ to 32^+6^ weeks gestational age.

Country	Received both magnesium sulfate and steroids (%)	Received magnesium sulfate only (%)	Received steroids only (%)	Received neither (%)	Total received magnesium sulfate (%)	Total received steroids (%)
High‐income						
UK	81.0	1.1	11.4	6.7	82.0	92.3
Ireland	82.3	1.3	10.8	5.7	83.6	93.1
Austria	71.7	0.9	20.6	6.8	72.7	92.5
Switzerland	74.9	1.1	20.0	4.0	76.0	95.1
Italy	53.3	0.7	36.8	9.2	53.9	90.3
US	73.3	2.4	14.5	9.8	75.7	87.7
UAE	42.9	1.5	42.2	13.4	44.5	85.1
Middle‐income						
Brazil	63.8	2.8	18.8	14.7	66.6	82.5
South Africa	31.9	1.7	40.7	25.7	33.6	73.1
India	50.0	0.2	30.8	18.9	50.0	81.0
**Mean for high‐income countries**	**72.6**	**2.3**	**15.6**	**9.6**	**74.8**	**88.1**
**Mean for middle‐income countries**	**47.3**	**2.1**	**30.2**	**20.3**	**49.4**	**77.8**

Abbreviation: VON, Vermont Oxford Network.

The proportion of infants receiving neither intervention also varied, from only 9.6% in these HICs (Ireland, the UK and Austria as the top‐performers all below 7%) compared to 20.3% in these MICs.

**FIGURE 1:**
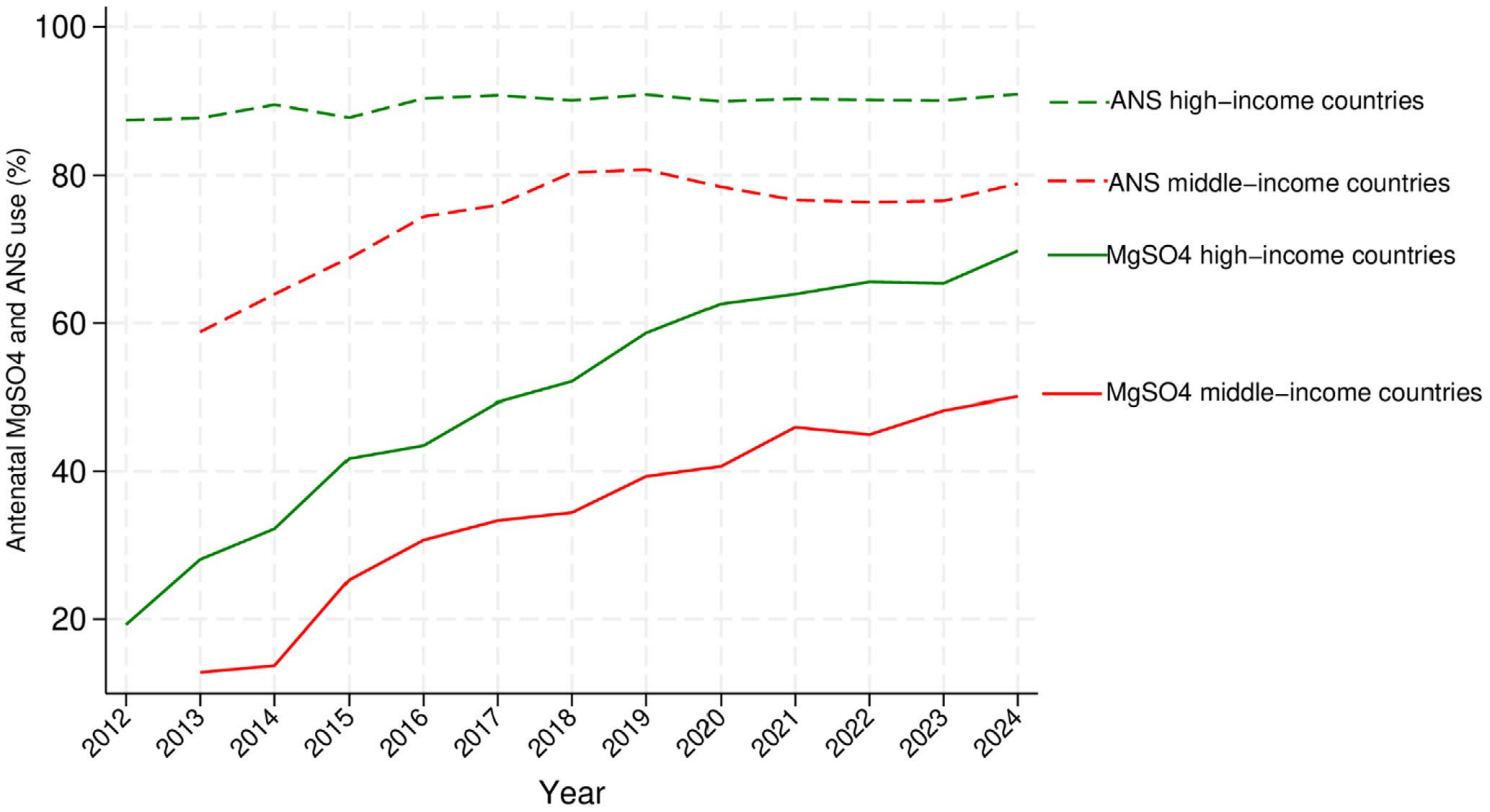
Trends in use of antenatal magnesium sulfate (MgSO_4_) and antenatal steroids (ANS) by income‐status.

Although uptake of both treatments has improved over time, the gap between these HICs and MICs appears to have sustained across the timeline of 2012 to 2024. For MgSO_4_ this treatment gap has sustained broadly around 20 percentage points difference. For ANS this treatment gap started at around 30 percentage points difference in 2013, then after some improvement in use in these MICs, sustained at around 10–12 percentage points difference between 2018 and 2014 (Figure 1, Appendix S3a,b).

Within countries, the countries with lower overall uptake of MgSO_4_ also tended to have more variation in use between centers. This association was less apparent in the case of ANS (Appendices 4a,b).

Use of both interventions was also broken down by GA subgroups (24–27 weeks; 28–30 weeks; 31–32 weeks). Across most of these countries, MgSO_4_ use tended to have a clear linear trend of decreasing use in older age groups (e.g., in the UK, 85.3% uptake at 24–27 weeks, 82.7% at 28–30 weeks, 70.6% at 31–32 weeks). Exceptions to this trend were the US, the UAE, and South Africa, where use was fairly consistent across all GA groups. ANS use was less clearly patterned by GA group, with most countries having uniformly high uptake across all ages (South Africa and India as possible exceptions with lower use in the 24–27 weeks' group) (Appendix S5).

### 3.4 Comparison of English data from VON and the NNRD

The relevant NNRD extract included data from 154 (out of total 155) centers in England, representing a population‐based database. A total of 17 of these were VON‐participating centers. The VON centers were largely representative of the wider NNRD population, with comparable proportions of multiples, cesarean deliveries, MgSO_4_ (84.1% vs. 82.6%) and ANS uptake (92.6% vs. 93.3%). One potential difference was that the GA and associated birth weight of infants in the VON centers appeared to be lower than the overall NNRD population (median GA 28 weeks, interquartile range [IQR: 26–30]) in VON, and 30 weeks (IQR: 28–32) in the NNRD; median birth weight 1029 g (IQR: 790–290 in VON, and 1364.5 g [IQR: 1020–680] in the NNRD). A second potential difference was that VON‐participating centers appeared to have a lower proportion of missing data on MgSO_4_ use (0.5% compared to 3.9% in NNRD data) (Appendix S6).

### 3.5 Supplementary review of the literature

A total of 183 records were identified from searches. These were pre‐sifted (by SD) and 114 removed as either ineligible or duplicate records. The remainder were title/abstract screened (by HE) and a further 20 excluded. A total of 49 full‐text reports were retrieved and assessed for eligibility (by HE). Of these, 10 were included in the supplementary review (Appendices 7, 8). Data extraction was performed (by HE) alongside the full‐text screening for eligibility.

Included reports (Appendix S8, Table 4) covered Canada (study IDs 1, 7), Australia and New Zealand (study IDs 2, 5, 6), the UK (study IDs 3, 8), Sweden (study ID 4), South America (study ID 9), and the USA (study ID 10). Three were national audit reports (study IDs 1, 2, 3), the others were cohort studies. In total there were 288 631 included infants from 22 to 33 weeks' gestation (age ranges varying between reports), although studies 3 and 8 contained overlapping populations. The audits reported on 2022 and 2023 data, which is comparable to the 2024 VON data, and the cohort studies had various study periods spanning 2009–2022 (all containing some data for 2015 or after).

**TABLE 4:** Supplementary literature review of data on use of antenatal magnesium sulfate.

Study ID	Country	Year of data	Data/study type	Number/type of centers	Number and gestational age of infants	Reported magnesium sulfate uptake (%)	Reported antenatal steroid uptake (%)
1	Canada	2023	National clinical network report	33 tertiary NICU centers	4278 <33 weeks; 1586 <29 weeks	82.2% <26 weeks; 85.8% 26–28 weeks; 76.8% 29–32 weeks	[90.1% <26 weeks];^a^ [91.1% 26–28 weeks]; [88.9% 29–32 weeks]
2	Australia and New Zealand	2022	National clinical network report	31 level III NICUs (25 Australia, 6 New Zealand)	[3294 24–31 weeks]; [4775 24–33 weeks]	59.5% 24–31 weeks	[85.4%] 24–33 weeks
3	UK (England, Scotland, Wales, the Isle of Man)	2023	National audit report	178 (54 NICU, 81 LNU, 43 SCBU)	[3915 <30 weeks];^b^ [11 768 23–33 weeks]	85.1% >30 weeks	[92.1% 23–33 weeks]^c^
4	Sweden	2021	Cohort study	Five university hospitals	388 22–32 weeks	78.6% [77.1% <28 weeks]; [80.6% 28–31 weeks]	Not reported
5	Australia and New Zealand	2012–2020	Binational registry study	Not reported	18 394 <30 weeks	78.8% in 2020	[82.3% across 2012–2020]
6	Australia and New Zealand	2016–2017	Cohort study	All centers in Victoria (number not reported)	244 23–<28 weeks	68.0%	88.7%
7	Canada	2011–2015	Cohort study	11 tertiary sites in the Canadian Perinatal Network	3143 24–32 weeks	48.1% 2011–2015	Not reported
8	UK (England, Scotland, Wales)	2014–2022	Cohort study	150 maternity units in England; 18 in Scotland; 12 in Wales	[3286 in England; 253 in Scotland; 135 in Wales <30 weeks]	85.5% in England; 81.4% in Scotland; 86.6% in Wales in 2022	[92.3% in England; 92.4% in Scotland; 92.6% in Wales in 2022]
9	South America	2015–2020	Cohort study	26 NEOCOSUR international neonatal network NICUs	7418 24–32 weeks	45.8% 2015–2020	85.5% 2015–2020
10	USA	2009–2018	Cohort study	NICUs contributing to the Pediatrix Clinical Data Warehouse (number not reported)	229 781 22–33 weeks	57.7% in 2018	87.4% in 2018

Abbreviations: LNU, local neonatal unit; NICU, neonatal intensive care unit; NNAP, National Neonatal Audit Programme; SCBU, special care baby unit.

^a^ Square brackets […] indicate calculated values, or values obtained from other sources (e.g., study authors), rather than directly reported in the source paper.

^b^ Numbers identified via NNAP data dashboard download.

^c^ Last reported for 2021.

Reported MgSO_4_ uptake varied both by study and by reported age group, the highest figures reported in the 2023 Canadian national audit (study ID 1), (85.8% for infants 26–28 weeks) and the 2022/2023 UK audit and cohort reports (study IDs 3, 8) (covering overlapping populations, with uptake at 86.6% in Wales, 85.5% in England, and 81.4% in Scotland for infants <30 weeks). The lowest figures came from earlier cohort studies in Canada (study ID 7) (48.1% uptake reported across 2011–2015 for infants 24–32 weeks), and in South America (study ID 9) (45.8% uptake reported across 2015–2020).

Two studies did not report ANS uptake, but of the remainder, 7/8 reported >85% uptake and three of these reported >90% uptake. The single study reporting <85% uptake (82.3%, study ID 5) reported figures as an average across 2012–2020. Overall, figures from both HICs and MICs found in the review were comparable to those reported in VON data (Table 4).

## 4 DISCUSSION

In this study, we report wide variation between countries in use of antenatal MgSO_4_ and ANS for preterm birth, despite these being evidence‐based and internationally recommended treatments. The most recent estimates show a gap in MgSO_4_ use between the HICs and MICs examined here of around 25 percentage points (around three quarters of eligible babies treated in HICs compared to around half in MICs). ANS were used more often than MgSO_4_ across all these countries, with less variation but still an observable treatment gap of around 10 percentage points (around 88% treated in these HICs compared to around 78% in these MICs). For MgSO_4_, there was additionally high variation in use even within the subgroup of HICs (54% in Italy and 44% in the UAE, compared to >80% in the UK and Ireland). The UAE appears to have a strikingly lower rate compared to the other HICs; however, due to the low number of UAE VON centers, including or excluding their results (a post hoc sensitivity analysis) makes negligible difference to the overall mean uptake for HICs. Within countries, those with lower overall uptake of MgSO_4_ also tended to have more variation in use between centers. Although uptake of both treatments appears to have improved over time in all included countries, the treatment gap between these HICs and MICs appears to have sustained across the timeline of this study (2012–2024).

The comparison of English VON data with NNRD data from all English NHS maternity units indicate that the VON data is generally representative, at least of other non‐VON hospitals in England. This representativeness may also apply across other nations, although we did not have data to check this. There was some indication that VON units might tend on average to have a younger, smaller, preterm population than other units in England (likely due to VON's inclusion criteria mainly focusing on younger and smaller babies). This does not appear to have translated into differences in rates of antenatal treatments, although it is possible that differences in care could emerge if patient populations were statistically adjusted for (e.g., in a weighted individual‐level analysis, which would be a valuable future study). VON units appeared to have slightly more complete treatment data, plausibly due to the additional quality control checks applied to data submitted to VON. However, the difference in missing data is small, so is unlikely to affect overall estimates of treatment rates.

Findings from the supplementary literature review also supported the overall picture from the VON data, with the most recent data on MgSO_4_ and ANS use from Canada, Sweden, Australia and New Zealand all broadly comparable to the HICs represented in the VON data, and use in South America comparable to the MICs represented in the VON data. There was some heterogeneity in the included supplementary studies, where unlike the VON centers, they may not all follow the same standard practice for use and reporting of antenatal MgSO_4_. The fact that despite this they gave a similar picture of a HIC/MIC gap in use to that seen in the VON data adds further weight to this finding.

The underlying reasons for the high variation in MgSO_4_ use in particular are unclear. Causal explanations cannot be inferred from this work, but we can speculate based on what else is known. For example, previous work has shown that antenatal interventions are difficult to implement,^31^ with QI work – which has only been undertaken in some countries – being a key factor in its success.^16^ Differences in patterning of births between countries could also affect uptake rates: for example, countries with a higher prevalence of home births and more rural populations, which may tend to be MICs, could reasonably be expected to have fewer opportunities to administer MgSO_4_.^32^ Additionally, MgSO_4_ is more challenging to administer than ANS, requiring more time to administer and with a shorter window of action before birth, challenges which again could disproportionately affect MICs.

It is less clear why MgSO_4_ use appears to vary so much even within HICs. Again, we can only speculate on possible reasons, but this could be due to factors including differences in healthcare systems, national guidelines,^4^ and perinatal QI efforts between countries. It is not likely to be a coincidence that the highest performing countries tend also to be the ones that have implemented formal, national QI programs for MgSO_4_ use,^13^, ^15^, ^16^ and that more consistency in use between centers within a country correlates with better overall rates. Future work should include causal exploration to improve understanding of these discrepancies.

A key strength of this study is that the data could be triangulated and thereby validated, with a consistent picture appearing across the three different data sources. The data is high‐quality and mainly routinely collected, which minimizes information bias. The NNRD data is nationally complete and thus fully generalizable across England, and VON data was found to be representative of the rest of the English data. Data from the supplementary review was nationally (or at least regionally) representative, again indicating generalizability. Another strength is the scale of the datasets – the VON data for 2024 alone reports on over 45 000 infants, with exponentially larger numbers across the historical data, enabling robust estimates of both current practice and time‐trends in use. Additionally, the present study reports on 2024 data, giving a highly current and up‐to‐date picture of international uptake of antenatal MgSO_4_ and ANS.

The limitations of the present study were that we did not have data on every country, and MICs and lower‐income countries were particularly underrepresented in this data. This indicates caution in generalizing from the MICS included here to other MICs. It is possible that MgSO_4_ and ANS use in other countries is different from the countries included here and could give a different picture of the variation by countries' income status. We speculate that the countries not included are likely, if anything, to have lower MgSO_4_ and ANS use than those represented here, as absence of reporting on these quality metrics is likely to correlate with lower performance on these same metrics. This suggests that the international disparities in care might be even greater than observed here.

A connected limitation is that VON is a voluntary, and so not population‐based network, meaning that VON hospitals do not necessarily represent or generalize to the rest of the population in those countries. We were able to confirm its representativeness of other English hospitals, and similar comparisons to check generalizability in other countries would be valuable future work.

The US is over‐represented in the data, as there are so many US VON centers. To help understand the impact of this weighting, we additionally calculated the mean uptake for HICs excluding the US (a post hoc sensitivity analysis). This slightly reduced the mean MgSO_4_ uptake for HICs from 74.8% to 67.8%, and slightly increased the mean ANS uptake for HICs from 88.1% to 91.2%. This slightly reduces the disparity in MgSO_4_ uptake between HICs and MICs, but the overall picture of a sizable treatment gap here remains.

As the data for this study was at the country‐level, we were not able to explore reasons for variation that might be clearer from individual‐level data. We had hoped to statistically compare MgSO_4_ uptake between HICs and MICs in the VON data, but the small number of countries made this analysis inappropriate. Moreover, given the clear group differences in the raw data, further statistical testing was arguably not warranted. Another limitation relates to the granularity of the data: MgSO_4_ is a dual‐use treatment, indicated for management of pregnancy hypertension and pre‐eclampsia, as well as neuroprotection. VON data does not distinguish the indication or timing of antenatal MgSO_4_, so some of the reported rates will have been boosted by the additional use for pre‐eclampsia (VON data on antenatal MgSO_4_ does exclude cases of postnatal use). VON data also does not distinguish between complete and incomplete courses of ANS, so it is possible that a different picture of care, and disparities in care, would emerge if we had additional data on ANS courses.

Future studies should ideally include data from more countries, more granular treatment data (e.g., indication, type, dose, timing in relation to birth, and course completeness), disparities in care at the individual as well as national‐level, causal exploration to improve understanding of the reasons behind low treatment rates in some countries, and where appropriate, extend roll‐out of the QI programs that have successfully improved treatment rates in others.

## 5 CONCLUSION

Despite being evidence‐based and internationally recommended treatments, antenatal MgSO_4_ and ANS use still vary considerably between, and within countries. On average less than half of eligible infants receive MgSO_4_ in the MICs evaluated here, and still only three‐quarters in the HICs evaluated here. Even within the HICs there are still large discrepancies in care. ANS is used more frequently with less variation, although treatment gaps are still apparent. While implementation of MgSO_4_ guidelines has occurred faster than that seen with ANS, recommendations have been clearer, and the reasons for delay unclear. Evidence is strong that improved antenatal optimization would mean lower rates of preventable life‐long disability, with associated improvements in individual and societal health, and economic costs. It would appear to be in interest of every nation to invest in and prioritize better preventative perinatal care.

## AUTHOR CONTRIBUTIONS

Karen Luyt conceptualized the study idea. Frank de Vocht, Erika M. Edwards, David Odd, Karen Luyt, and Hannah B. Edwards all contributed to the study planning and methodology. Aggregate VON data was contributed by Erika M. Edwards, who is the data guarantor. Mark Adams, Angelika Berger, Paul Corcoran, Jose Maria de Andrade Lopes, Luigi Gagliardi, R. Kishore Kumar, Rajesh Sharma, Lloyd Tooke, Erika M. Edwards and Karen Luyt were responsible for the collection of VON data in the included countries and gave their permission for their aggregate data to be used in this study. Jelena Savović and Sarah Dawson advised on the supplementary literature search strategy, and Sarah Dawson performed the searches and initial prescreening. Screening and data collection for the literature review was performed by Hannah B. Edwards. Analysis and interpretation of all data was performed by Hannah B. Edwards with input from all co‐authors. The manuscript was written by Hannah B. Edwards with input from all co‐authors. All authors approved the decision to submit for publication. Frank de Vocht and Karen Luyt were co‐chief Investigators.

## FUNDING INFORMATION

This research was funded by the National Institute for Health and Care Research Applied Research Collaboration West (NIHR ARC West). The views expressed in this article are those of the author(s) and not necessarily those of the NIHR or the Department of Health and Social Care.

## CONFLICT OF INTEREST STATEMENT

Erika M. Edwards is Chief Scientific Officer and Director of Data Science at Vermont Oxford Network. The authors report no conflicts of interest.

## Supporting information


**Data S1:** Supplementary material.

**Data S2:** STROBE_checklist_cohort V2.

## Data Availability

VON and NNRD data dictionaries are publicly available at https://public.vtoxford.org/ and https://digital.nhs.uk/data‐and‐information/information‐standards/governance/latest‐activity/standards‐and‐collections/dapb1595‐neonatal‐data‐set/. These data are accessible via formal application to these organizations.
