# Supplementary material for: International disparities in use of antenatal magnesium sulfate and antenatal steroids for the preterm baby
Source: Int J Gynaecol Obstet. 2026 Feb 25;174(2):707–17. doi: 10.1002/ijgo.70832 (PMC13377267; doi:10.1002/ijgo.70832)
Supplement: Supplementary file 1 — Data S1: Supplementary material. [file IJGO-174-707-s002.docx]

**Supplementary material**

**Appendix 1: Inclusion criteria for supplementary review**

Format: scoping the literature

Inclusion criteria for audit reports (grey literature):

- Audit reports
- National-level
- Data within the last 5 years (2020 to 2025)
- High-income European countries
- Contains data on MgSO_4_ exposure for infants 24-32 weeks gestation
- Reported figures based on individual-level data

Inclusion criteria for standard journal articles:

- QI reports, cohort studies, cross-sectional studies, surveys
- National or regional-level
- Data within the last 10 years (2015 to 2025)
- Contains data on MgSO_4_ exposure for infants 24-32 weeks GA
- Reported figures based on individual-level data

Risk of bias assessment: Not indicated, as this is not an evaluation of evidence for effectiveness.

**Appendix 2: Example of search strategy**

| Ovid MEDLINE(R) ALL <1946 to August 06, 2025>  Updated 10-Sept-2025, new records, n=2 | | |
| --- | --- | --- |
| 1 | magnesium compounds/ or magnesium sulfate/ | 8410 |
| 2 | (magnesium adj (sulphate or sulfate)).mp. | 8843 |
| 3 | "MgSO4".mp. | 3141 |
| 4 | or/1-3 | 13283 |
| 5 | Neuroprotection/ or Neuroprotective Agents/ | 49097 |
| 6 | (neuroprotect* or (neur* adj2 protect*)).mp. | 120711 |
| 7 | (cerebral pals* or infantilecerebral pals* or little* disease or spastic diplegi* or congenitalcerebral pals*).mp. | 35723 |
| 8 | or/5-7 | 155910 |
| 9 | 4 and 8 | 747 |
| 10 | obstetric labor, premature/ or premature birth/ | 37440 |
| 11 | infant, premature/ or infant, extremely premature/ | 70047 |
| 12 | ((prem* or preterm* or pre-term*) adj3 (birth* or labo?r)).mp. | 69431 |
| 13 | ((prem* or preterm* or pre-term*) adj3 (baby or babies or infant* or neonat* or born or newborn)).mp. | 115041 |
| 14 | (perinat* or peri-nat*).mp. | 107057 |
| 15 | Prenatal Care/ | 34865 |
| 16 | ((prenatal or antenatal) adj (care or healtcare)).mp. | 52054 |
| 17 | or/10-16 | 292428 |
| 18 | 9 and 17 | 423 |
| 19 | birth cohort/ | 1626 |
| 20 | cohort studies/ or follow-up studies/ | 1034353 |
| 21 | "Surveys and Questionnaires"/ | 633452 |
| 22 | (longitudinal stud* or cohort? or followup or follow-up).mp. | 2813653 |
| 23 | (survey* or questionnaire*).mp. | 1793612 |
| 24 | Registries/ | 120356 |
| 25 | (register or registers or registry or registries).mp. | 344013 |
| 26 | (data and (national or regional)).mp. | 504209 |
| 27 | ((chart or charts) adj1 (patient* or medical* or review*)).mp. | 76164 |
| 28 | ((prem* or neonat*) adj3 database*).mp. | 1751 |
| 29 | clinical audit/ or medical audit/ | 19733 |
| 30 | (audit or audits).mp. | 63892 |
| 31 | guideline*.mp. | 705256 |
| 32 | or/19-31 | 5329327 |
| 33 | 18 and 32 | 161 |
| 34 | limit 33 to yr="2015 -Current" | 120 |
| 35 | ("28903747" or "33528040" or "31119725" or "37680134" or "39821226" or "40288887" or "29325592").ui. | 7 |
| 36 | 34 and 35 | 7 |

**Appendix 3a: Trends in use of antenatal magnesium sulphate (MgSO4) by country**

**
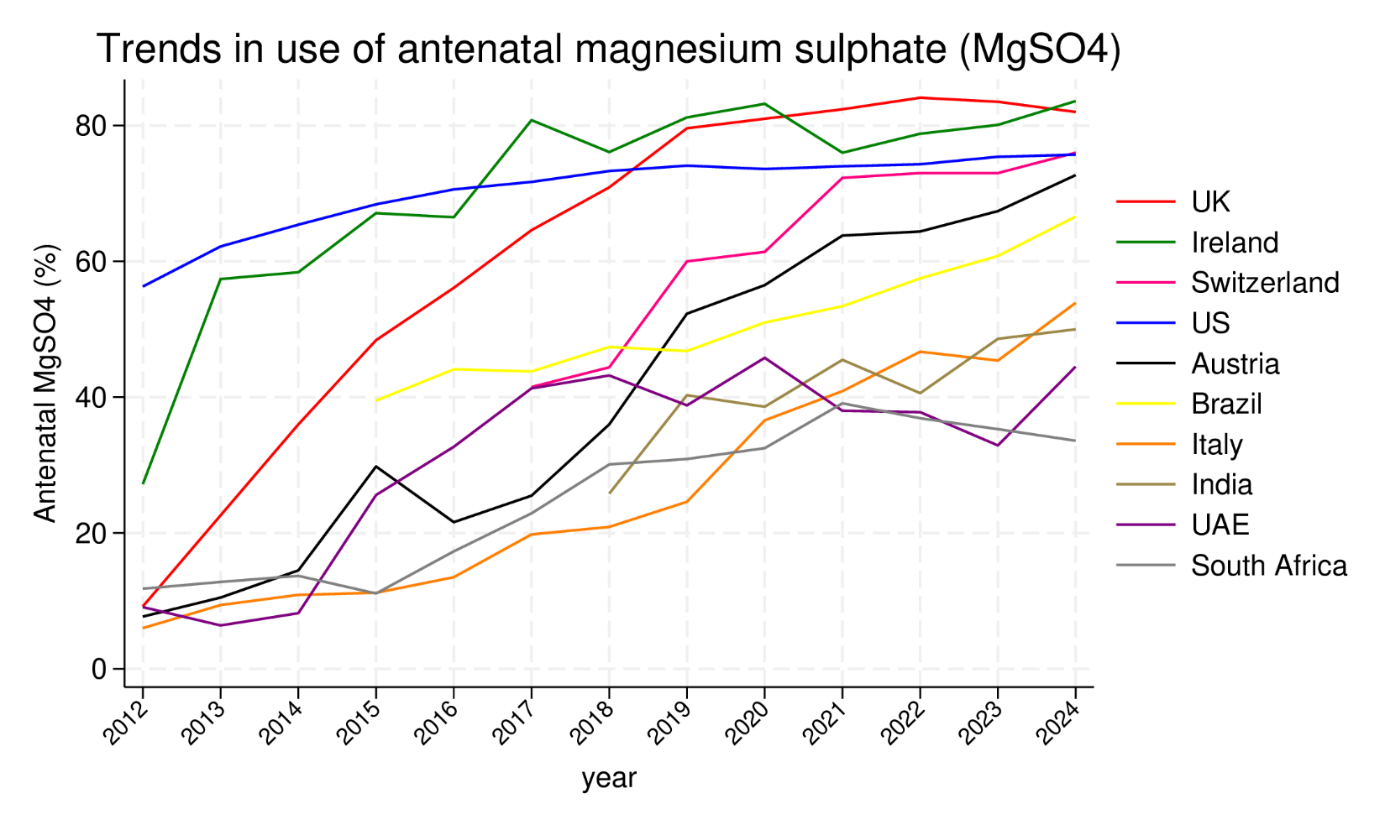
**

**Appendix 3b: Trends in use of antenatal steroids (ANS) by country**


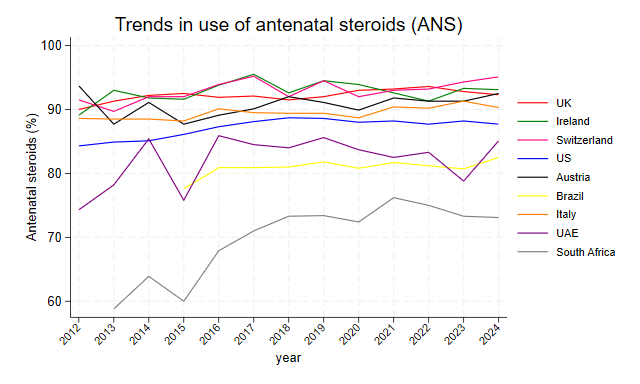


**Appendix 4a: Variation in use of antenatal magnesium sulphate (MgSO4) by country**

**Appendix 4b: Variation in use of antenatal steroids (ANS) by country**

**Appendix 5: Exposure to antenatal interventions in Vermont Oxford Network data for 2024, by gestational age group**

| **Country** | **Received magnesium sulphate (%)** | | | | **Received steroids**  **(%)** | | | |
| --- | --- | --- | --- | --- | --- | --- | --- | --- |
|  | **Overall** | **24-27 weeks** | **28-30 weeks** | **31-32 weeks** | **Overall** | **24-27 weeks** | **28-30 weeks** | **31-32 weeks** |
| *High-income* |  |  |  |  |  |  |  |  |
| UK | 82·0 | 85·3 | 82·7 | 70·6 | 92·3 | 91·9 | 91·0 | 96·4 |
| Ireland | 83·6 | 88·2 | 87·6 | 62·7 | 93·1 | 89·8 | 94·7 | 95·6 |
| Austria | 72·7 | 82·7 | 78·5 | 36·0 | 92·5 | 96·0 | 92·0 | 85·9 |
| Switzerland | 76·0 | 81·4 | 80·4 | 49·3 | 95·1 | 95·7 | 93·9 | 97·2 |
| Italy | 53·9 | 61·7 | 53·2 | 45·2 | 90·3 | 90·1 | 90·2 | 90·9 |
| US | 75·7 | 76·9 | 77·5 | 68·9 | 87·7 | 86·2 | 88·1 | 90·2 |
| UAE | 44·5 | 42·4 | 45·4 | 46·2 | 85·1 | 82·2 | 95·2 | 90·2 |
| *Middle-income* |  |  |  |  |  |  |  |  |
| Brazil | 66·6 | 68·7 | 69·0 | 58·4 | 82·5 | 82·3 | 83·0 | 82·1 |
| South Africa | 33·6 | 31·5 | 34·0 | 35·3 | 73·1 | 64·2 | 77·2 | 75·8 |
| India | 50·0 | 62·7 | 51·9 | 34·5 | 81·0 | 79·4 | 94·2 | 80·0 |
| **Mean for high-income countries** | 74·8 | 76·5 | 76·6 | 67·0 | 88·1 | 86·7 | 88·4 | 90·5 |
| **Mean for middle-income countries** | 49·4 | 51·7 | 49·5 | 45·4 | 77·8 | 74·0 | 80·0 | 79·0 |

**Appendix 6: Comparison of English data from the Vermont Oxford Network and the UK National Neonatal Research Database for 2024, infants 24^+0^ to 32^+6^ weeks gestational age**

|  | **VON** | **NNRD** |
| --- | --- | --- |
| Number of centres | 17 | 154 |
| Number of eligible babies | 950 | 7742 |
| Gestational age, weeks (median, IQR) | 28 (26, 30) | 30 (28, 32) |
| Birthweight, grams (median, IQR) | 1029 (790, 1290) | 1364·5 (1020, 1680) |
| Multiple (%) | 25·4 | 23·4 |
| Caesarean delivery (%) | 73·3 | 72·6 |
| Reported magnesium sulphate uptake (%) | 84·1 | 82·6 |
| Missing magnesium sulphate data (%) | 0·5 | 3·9 |
| Reported antenatal steroid uptake (%) | 92·6 | 93·3 |
| Missing antenatal steroid data (%) | 0·0 | 0·5 |

Data includes all multiples

**Appendix 7: PRISMA flow diagram**

**Identification of studies via databases and registers**

Records removed *before screening*:

Duplicates (n = 21)

Pre-sifted/ineligible (n = 93)

Records identified from:

MEDLINE (n = 121)

CINAHL (n = 42)

Google (n = 12)

Citation Chaser (n=8)

Total (n = 183)

**Identification**

Records excluded

(n = 20)

Records screened (n = 69)

Reports not retrieved

(n = 0)

Reports sought for retrieval

(n = 49)

**Screening**

Reports excluded: (n = 39)

- Did not report individual level data (n = 3)
- Single-centre study (n = 10)
- Case-control or case report (n = 2)
- Lab study (n = 1)
- Qualitative data only (n = 2)
- RCT data only (n = 8)
- Evaluation of KT methods (n = 1)
- Review of guidelines (n = 4)
- Overview paper (n = 2)
- More recent data available on the same cohort (n = 1)
- Pre-2015 data only (n = 5)

Reports assessed for eligibility

(n = 49)

Studies/reports included in review (n = 10)

**Included**

**Appendix 8: References included in supplementary review**

| **ID** | **Reference** |
| --- | --- |
| **1** | The Canadian Neonatal Network 2023 Annual Report  <https://www.canadianneonatalnetwork.org/portal/Portals/0/Annual%20Reports/2023%20CNN%20Annual%20Report.pdf> |
| **2** | Report of the Australian and New Zealand Neonatal Network 2022  <https://anznn.net/Portals/0/AnnualReports/Report%20of%20the%20Australian%20and%20New%20Zealand%20Neonatal%20Network%202022.pdf> |
| **3** | National Neonatal Audit Programme (NNAP) 2023 Data: Extended Analysis Report  <https://www.rcpch.ac.uk/sites/default/files/2024-10/nnap_2023_data_extended_analysis_report_v2.pdf> |
| **4** | A follow up on the feasibility after national implementation of magnesium sulfate for neuroprotection prior to preterm birth. Hellström S et al. AOGS September 2023  <https://obgyn.onlinelibrary.wiley.com/doi/10.1111/aogs.14673> |
| **5** | Increasing Use of Antenatal Magnesium Sulphate Prior to Preterm Birth for Preventing Cerebral Palsy in Australia and New Zealand, 2012–2020: A Binational Registry Study. Shepherd et al. ANZJOG January 2025  <https://obgyn.onlinelibrary.wiley.com/doi/10.1111/ajo.13937> |
| **6** | Translating antenatal magnesium sulphate neuroprotection for infants born <28 weeks' gestation into practice: A geographical cohort study. Doyle et al. ANZJOG February 2021  <https://obgyn.onlinelibrary.wiley.com/doi/10.1111/ajo.13301> |
| **7** | MAGnesium sulphate for fetal neuroprotection to prevent Cerebral Palsy (MAG-CP)-implementation of a national guideline in Canada. De Silva DA et al. Implement Sci. 2018;13(1):8.  <https://pmc.ncbi.nlm.nih.gov/articles/PMC5765609/> |
| **8** | Implementation of national guidelines on antenatal magnesium sulfate for neonatal neuroprotection: extended evaluation of the effectiveness and cost-effectiveness of the National PReCePT Programme in England. Edwards HB et al. BMJ Qual Saf. 2025;27:27.  <https://qualitysafety.bmj.com/content/early/2025/04/24/bmjqs-2024-017763.long> |
| **9** | Antenatal exposure to magnesium sulfate and neonatal outcomes in very low birth weight infants: a multicenter study. Vaz Ferreira C et al. NEOCOSUR Neonatal Network. J Perinatol. 2024 Nov;44(11):1663-1668.  <https://www.nature.com/articles/s41372-024-02025-y> |
| **10** | Changing Tocolytic Exposures among Neonatal Intensive Care Unit Admitted Preterm Infants. Jarman ML etal. Am J Perinatol. 2022;39(16):1745-9.  <https://www.thieme-connect.de/products/ejournals/abstract/10.1055/a-1745-3262> |
